# Where to go to in chlamydia control? From infection control towards infectious disease control

**DOI:** 10.1136/sextrans-2021-054992

**Published:** 2021-05-27

**Authors:** Jan E A M van Bergen, Bernice Maria Hoenderboom, Silke David, Febe Deug, Janneke C M Heijne, Fleur van Aar, Christian J P A Hoebe, Hanna Bos, Nicole H T M Dukers-Muijrers, Hannelore M Götz, Nicola Low, Servaas Antonie Morré, Bjőrn Herrmann, Marianne A B van der Sande, Henry J C de Vries, Helen Ward, Birgit H B van Benthem

**Affiliations:** 1 Department General Practice/Family Medicine, Amsterdam UMC Location AMC, Amsterdam, The Netherlands; 2 STI AIDS Netherlands, Amsterdam, The Netherlands; 3 Centre for Infectious Disease Control, National Institute for Public Health and the Environment (RIVM), Bilthoven, The Netherlands; 4 Department of Social Medicine and Medical Microbiology, Care and Public Health Research Institute (CAPHRI), Maastricht University, Faculty of Health, Medicine and Life Sciences, Maastricht, The Netherlands; 5 Department Sexual Health, Infectious Diseases and Environmental Health, Public Health Service South Limburg, Heerlen, The Netherlands; 6 Department of Health Promotion, Care and Public Health Research Institute (CAPHRI), Maastricht University, Maastricht, The Netherlands; 7 Department of Sexual Health, Infectious Diseases, and Environment, Public Health Service South Limburg, Heerlen, The Netherlands; 8 Department of Infectious Disease Control, Rotterdam Rijnmond Public Health Services, Rotterdam, The Netherlands; 9 Institute of Social and Preventive Medicine, University of Bern, Bern, Switzerland; 10 Institute for Public Health Genomics, Genetica & Cell Biology, Maastricht University Faculty of Health Medicine and Life Sciences, Maastricht, The Netherlands; 11 Dutch Chlamydia trachomatis Reference Laboratory, National Institute for Public Health and the Environment, Bilthoven, The Netherlands; 12 Department of Clinical Microbiology, Uppsala University Hospital, Uppsala, Sweden; 13 Department of Public Health, Institute of Tropical Medicine, Antwerp, Belgium; 14 Global Health, Julius Center, Utrecht University, Utrecht, The Netherlands; 15 Department of Infectious Diseases, Public Health Service Amsterdam, Amsterdam, The Netherlands; 16 Department of Dermatology, Academic Medical Center, Amsterdam, The Netherlands; 17 Infectious Disease Epidemiology, Imperial College London, London, UK

**Keywords:** chlamydia Infections, diagnostic screening programs, health services research, infection control, pelvic inflammatory disease

## Abstract

**Objectives:**

The clinical and public health relevance of widespread case finding by testing for asymptomatic chlamydia infections is under debate. We wanted to explore future directions for chlamydia control and generate insights that might guide for evidence-based strategies. In particular, we wanted to know the extent to which we should pursue testing for asymptomatic infections at both genital and extragenital sites.

**Methods:**

We synthesised findings from published literature and from discussions among national and international chlamydia experts during an invitational workshop. We described changing perceptions in chlamydia control to inform the development of recommendations for future avenues for chlamydia control in the Netherlands.

**Results:**

Despite implementing a range of interventions to control chlamydia, there is no practice-based evidence that population prevalence can be reduced by screening programmes or widespread opportunistic testing. There is limited evidence about the beneficial effect of testing on pelvic inflammatory disease prevention. The risk of tubal factor infertility resulting from chlamydia infection is low and evidence on the preventable fraction remains uncertain. Overdiagnosis and overtreatment with antibiotics for self-limiting and non-viable infections have contributed to antimicrobial resistance in other pathogens and may affect oral, anal and genital microbiota. These changing insights could affect the outcome of previous cost–effectiveness analysis.

**Conclusion:**

The balance between benefits and harms of widespread testing to detect asymptomatic chlamydia infections is changing. The opinion of our expert group deviates from the existing paradigm of ‘test and treat’ and suggests that future strategies should reduce, rather than expand, the role of widespread testing for asymptomatic chlamydia infections.

## Introduction

Chlamydia control strategies are based on the prevailing infectious disease paradigm that control of STIs is achieved by reducing the reproduction number (R_0_) of *Chlamydia trachomatis*, the bacterium that causes chlamydia infection. The formula R_0_=βcD shows that the reproduction number can be reduced by preventing acquisition (reducing transmission efficiency β), limiting the partner change rate (c) and/or by reducing the duration of infectiousness (D). Behavioural interventions, such as increasing correct and consistent condom use, are primary prevention interventions to reduce transmission. Testing and treatment are secondary prevention interventions that aim to reduce the duration of infectiousness (D). As most chlamydia infections are asymptomatic, finding and treating these infections start with testing and case finding.

**Figure 1:**
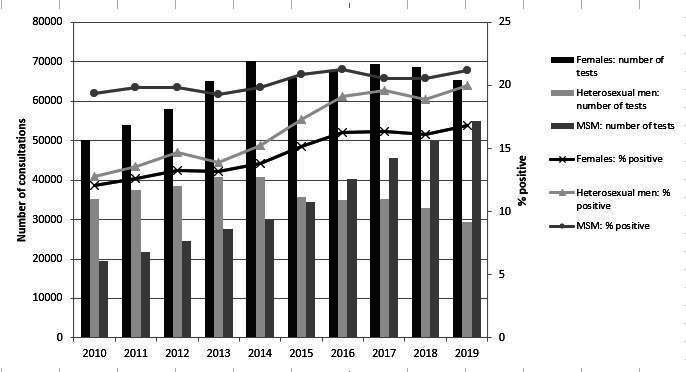
Total number of tests and positivity rate of chlamydia by gender and type of sexual contact, 2010–2019 Sexual Health Centres in the Netherlands.^2^ MSM, men who have sex with men.

Early diagnosis and treatment should prevent onward transmission and individual adverse health outcomes. For HIV infection, this ‘test and treat’ strategy has proven to be a very effective and successful public health intervention. In the Netherlands, the number of annual new HIV diagnoses reduced by 72% in the last 10 years.^1^ However, no such trend exists for chlamydia, despite widespread opportunistic testing for asymptomatic infections (figure 1).^2^

The Dutch National Action Plan on STI, HIV and Sexual Health 2017–2022 recommended an update of the strategies to prevent the burden of disease caused by infections with *C. trachomatis* (‘chlamydia’).^3^

In November 2019, an invitational workshop brought together a range of national and international chlamydia experts to explore future avenues for chlamydia control in the Netherlands (see online supplemental file 1). In particular, we wanted to know the extent to which we should pursue testing for asymptomatic infections at both genital and extragenital sites. In this paper, we describe our problem analysis and the main outcome of the workshop.

10.1136/sextrans-2021-054992.supp1 Supplementary data

## Methods

To capture recommendations about how imperfect evidence might be translated into a roadmap for the near future in the Netherlands, we chose to draw on expert opinion collated during a meeting that included the authors of this paper. The expert meeting was preceded by a narrative review of reviews and relevant articles (JvB), synthesised and presented during the 2019 International Union Against STI Europe conference^4^ and presented at the start of the expert meeting. We also used as background the external review evidence pack of the English National Chlamydia Screening Programme External Peer Review.^5^ We asked meeting participants if their perspectives on chlamydia control had changed over the preceding 10 years and discussed the evidence for these changes. We elaborated on views and interpretation of available evidence about organised screening programmes (referred to as screening) and on opportunistic testing of asymptomatic persons in healthcare settings (referred to as routine testing) and discussed the impact of this widespread testing, including out-of-clinic testing and internet testing for chlamydia infections.

Our debates covered heterosexual persons, men who have sex with men (MSM) and pregnant women (online supplemental file 2). The problem analysis and the consensus viewpoint that evolved from this expert meeting showed a shift from the existing ‘test and treat’ paradigm in chlamydia control and suggests that future strategies should reduce, rather than expand, the role of widespread testing for asymptomatic chlamydia infections.

10.1136/sextrans-2021-054992.supp2 Supplementary data

### Problem analysis

Early detection and treatment aim to limit onward spread and prevent reproductive health sequelae in women. New developments in point-of-care tests, mobile health, home sampling and internet testing offer increasing opportunities to implement widespread testing to detect asymptomatic chlamydia infections, including extragenital infections.^6–8^ However, the clinical and public health relevance of active case finding by testing for asymptomatic infections is under debate.^9–11^ The balance between benefits and harms of testing for asymptomatic infections might be changing for three reasons: (1) empirical evidence does not support the claim that enhanced testing of asymptomatics reduces the incidence and prevalence of chlamydia infection; (2) the risk of long-term complications attributable to a chlamydia infection in particular the risk of tubal factor infertility (TFI) is considered low and there is uncertainty about the fraction that can be prevented by routine testing; (3) there is more acknowledgement of the harms related to overdiagnosis and overtreatment, including the potential for antimicrobial resistance in other pathogens.

#### Lack of evidence for prevalence reduction

In 2018, 26 European Union/European Economic Area member states reported 406 406 confirmed cases of chlamydia infection. The overall notification rate of confirmed cases between 2009 and 2018 remained rather stable in countries reporting consistently, including countries recommending widespread testing like the UK and the Scandinavian countries.^12^ Surveillance data however relate to the amount of testing in a country and who is being tested. They do not reflect population prevalence: ecological associations do not provide evidence of effectiveness of interventions and may even lead to ‘ecological fallacies’.^13^ Research from pragmatic randomised controlled trials and observational studies of chlamydia prevalence has accumulated in the last decade. That evidence suggests that real-life screening programmes or widespread opportunistic testing is unlikely to reduce prevalence. The chlamydia screening implementation programme in three regions in the Netherlands concluded that, at the achieved levels of uptake, there was no statistical evidence of an impact on chlamydia positivity rates or estimated population prevalence after 3 years of a regular postal invitation to all persons aged 16–29 years to take a sample at home and mail it for a screening test for *C. trachomatis*. As a result, the Dutch government decided not to implement a national roll-out of register-based chlamydia screening.^9 10^ The National Chlamydia Screening Program in England has driven large increases in chlamydia testing since 2003, testing in the last decade more than 1 million young people aged 15–24 years, including up to 30% of all young women.^14^ Although previous modelling studies estimated that these levels of uptake should be sufficient to reduce population prevalence, two population-based surveys, done as part of the British National Surveys of Sexual Attitudes and Lifestyles, found that the prevalence estimates were compatible with no decrease in chlamydia prevalence between 2000 and 2011.^15^ The National Chlamydia Screening Program in England conducted an internal and external review and is now reframing its priorities.^5^ In Australia, the Australian Chlamydia Control Effectiveness Pilot, a cluster randomised controlled trial from 2010 to 2014, concluded that a sustained and relevant uptake of widespread testing was not feasible and that sizeable reductions in chlamydia prevalence are unlikely to be achieved.^16^

#### Low rates of severe long-term complications

Potential short-term and long-term complications for women are pelvic inflammatory disease (PID) and compromised fertility (TFI and risk of ectopic pregnancy) and chronic pelvic pain. For PID, it is difficult to obtain reliable data because most diagnoses are based on clinical signs and symptoms only, there is no accepted gold standard for diagnosis and the concept of ‘silent’ PID contributes to diagnostic uncertainty. A multiparameter evidence synthesis, which uses statistical modelling informed by empirical data, estimated the population attributable fraction of chlamydia infection in PID to be around 20%–30%.^17^ There is limited, low to moderate quality evidence from randomised controlled trials that testing asymptomatic women reduces the risk of PID, and the trials at lower risk of bias show smaller reductions than those at higher risk of bias.^18^ PID can easily be treated if symptoms develop and the major goal behind testing and treating women with asymptomatic chlamydia infection is the potential impact of both symptomatic as well as subclinical and silent PID on female fertility (TFI).^19^ However, there is a paucity of evidence that widespread testing indeed will reduce TFI. A crucial knowledge gap is uncertainty at what moment in time tubal damage occurs in the natural course of infection, and whether treatment of asymptomatic cases can be in time to prevent adverse outcomes once the infection is detected. Tubal tissue damage might already have occurred between acquisition of the infection and the detection, which might partly explain why tested (and treated) women have higher complication rates compared with those not tested.^20–22^

Realistic estimates of the preventable fraction of adverse fertility outcomes are difficult to obtain as observational studies are fraught with bias.^22^ There are no controlled studies using TFI and ectopic pregnancy as outcome measure. The long time lapse between infection and planned pregnancy makes such trials very difficult and therefore we depend on modelling. A multiparameter evidence synthesis estimated that 1000 chlamydia infections in women will lead to five cases of TFI and two cases of ectopic pregnancy.^18^ In an analysis of a Dutch cohort study of women with and without a history of chlamydia infection, we observed only a slight difference in time to pregnancy, and overall no difference in the proportion getting pregnant, suggesting that the effect of chlamydia control in preventing late complications that result in infertility might be very small.^23^

Other potential reproductive health complications of a chlamydia infection are adverse pregnancy outcomes like preterm birth, low birth weight and postpartum infections in mother and/or newborn.^24 25^ Although many observational studies show these associations, controlled studies are needed to demonstrate that screening and treating asymptomatic chlamydia infections during pregnancy can reduce these adverse outcomes. For bacterial vaginosis (BV), similar associations with preterm birth and low birth weight have been reported consistently, but studies have repeatedly shown no net benefits of BV testing and treating of pregnant women, and the US Preventive Services Task Force now recommends against BV screening in pregnant persons.^26^

For men, complication rates of chlamydia infections—excluding *Lymfogranuloma venereum*—are low, and most infections are asymptomatic and self-limiting. An important argument to test for asymptomatic chlamydia infection in MSM, including rectal infections, was the cofactor effect of other STIs in the transmission of HIV. However, the impact of this effect at the population level currently in high-income countries is likely to be small. In 2020 in the Netherlands, 93% of MSM were estimated to be aware of their HIV status, 95% of them was on treatment and 97% of those had an undetectable viral load.^1^ Meanwhile, the roll-out of pre-exposure prophylaxis (PrEP) in HIV-negative men at risk will continue to reduce HIV incidence. In the current phase of the HIV epidemic in the Netherlands, chlamydia testing among asymptomatic MSM is unlikely to have major impact on numbers of averted new HIV infections. Still it is common practice in the Netherlands to test routinely for chlamydia in asymptomatic MSM up to four times a year (if they use PrEP) on three locations: urogenital, oral and rectal.

#### Potential harms from overdiagnosis and overtreatment

A number of potential harms have been reported in the past. A diagnosis of chlamydia can induce feelings of dirtiness and stigma, lead to relationship break-ups and cause anxiety about compromised fertility.^27^ Negative test results might reinforce unsafe sexual practices.^28^ A potential harm of widespread testing at the population level is framed in the arrested immunity hypothesis: early detection and treatment of infections might abort immune responses and render individuals more vulnerable for reinfection, potentially leading to more severe sequelae.^29^ Although this arrested immunity remains a hypothesis, partial immunity and substantial reduction in susceptibility against reinfection was found necessary in mathematical models to explain observed chlamydia prevalence.^30^

In recent years, more attention is being paid to the health consequences of overdiagnosis and overtreatment. Overdiagnosis is defined as overdetection of a medical condition that would never have led to symptoms, disease or death.^31^ Although the aim is to improve health, overdiagnosis of conditions in people who are asymptomatic can lead to psychological stress and harmful interventions.^32^ Detecting and treating asymptomatic chlamydia infections might qualify for this line of reasoning. The vast majority of genital chlamydia infections are asymptomatic and self-limiting not leading to symptoms or disease. For genital infections, the median spontaneous clearance without treatment is around 1 year.^17 33^ Oral chlamydia infections are rare and asymptomatic, and spontaneous clearance rate between detection and the start of treatment is much more substantial. In a recent study, 50% of infections had already resolved after 9-day follow-up without yet having received any treatment.^34^ Anal chlamydia infections in MSM and women are mostly asymptomatic and unrelated to reported anal sexual behaviour. Their natural history is poorly understood, both in terms of their pathogenicity as in their role in genital reinfection in women.^34 35^

A significant proportion of the detected extragenital infections detected by nucleic acid amplification tests appears to be non-viable.^36^ In oral chlamydia samples, percentage viability is reported only around in 26% and in anal samples around 60%, compared with 95% in genital samples. Non-viable chlamydia will not impact clinical outcome nor transmission.^34 36 37^

Testing for asymptomatic infections means that test-positive individuals and their, often untested and asymptomatic, partners are treated with antibiotics although the majority will never develop either symptoms or complications. The number of unnecessary treated persons is much higher for chlamydia infection than for STI like HIV or syphilis. In the case of HIV, nearly all infected people will develop AIDS and die if left untreated. One-third of untreated syphilis cases will develop late complications. In contrast, for chlamydia, the risk for women to develop TFI is estimated less than 1%, meaning that 99% of these women will be treated with antibiotics unnecessarily for this indication. Moreover, as explained, we do not know if treatment will even be in time to prevent this 1%.

Overtreatment with antibiotics in asymptomatics contributes to increased antimicrobial resistance (AMR). AMR is considered by the WHO as a major global health threat requiring strict antibiotic stewardship. AMR is, for now, not so much a problem for the *C. trachomatis*, which remains susceptible to standard antibiotic regimens. But widespread treatment with broad-spectrum antimicrobials can result in emergence of AMR for other STI and non-STI pathogens. In particular, overtreatment is of concern in MSM communities with high interconnectivity, and with frequent testing and treatment of STI, for example, during PrEP monitoring.^38^ Last but not least, antibiotic treatment affects oral, vaginal and rectal microbiota. A healthy microbiome is considered a major factor in the prevention of infections and reinfection.^39^

## Discussion

**Figure 2:**
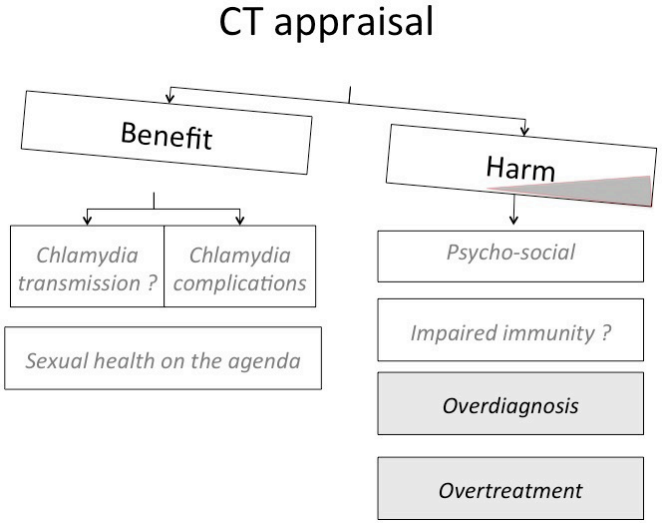
Chlamydia appraisal: shifting balance between benefits and harms.

The expert group concluded that the changing body of evidence urges a re-evaluation of the benefit-to-harm ratio and the cost-effectiveness of widespread testing for asymptomatic chlamydia infections in high-income countries. The consensus of the expert group was that the balance between benefits and harms is shifting (figure 2). On the benefit side, there is a potential benefit in PID prevention, but high-quality empirical evidence for realising better fertility and pregnancy outcomes is absent. Meanwhile, there is evidence that the recommendations for widespread opportunistic testing or screening in high-income countries have not substantially reduced chlamydia prevalence in the real world. On the side of potential harms, the overdiagnosis of self-limiting and non-viable infections has led to overuse of antimicrobials, which contributes to AMR in other pathogens and affects microbiota.

These insights should be incorporated in future modelling of the impact of chlamydia interventions and the outcomes might differ from those of previous model-based evaluations of costs and benefits. They raise the question whether widespread opportunistic testing for asymptomatic chlamydia is good value for money amidst increasing healthcare costs in a restrained economic situation.

Considering the limited and conflicting evidence currently available, we do not recommend age-based screening and widespread testing for chlamydia in asymptomatic persons in the Netherlands. Instead, we see targeted testing combined with effective management strategies (appropriate treatment and partner notification) and prevention (eg, behavioural counselling) as a way forward. This targeted strategy we define as ‘infectious disease control’ and focuses on harm reduction, in contrast to a ‘test and treat strategy’ that aims to detect as much infections as possible (‘infection control’).^4^

New developments, including genetic profiling, immunogenetics, biomarkers and diagnostic innovations to characterise the infection (viability PCR/bacterial load), are expected to be helpful for this targeted and tailored strategy in the nearby future.

If scaling down widespread testing for asymptomatic chlamydia infections in the Netherlands is adopted, this will need strategic planning, co-creation with communities and careful monitoring, with an explicit agenda for research and evaluation as this approach deviates from earlier policies. In recent decades, health authorities have promoted just the opposite: widespread chlamydia testing has been incorporated in national guidelines, and public campaigns in the media have informed the public that chlamydia is the number one STI.

To change existing perceptions and practices, even with newly emerging and growing evidence, is not an easy task. Doing less rather than more is even more difficult to ‘sell’. Moreover, there are strong lobby and profit-oriented interests that benefit from the promotion of testing, including those of diagnostic companies and for-profit commercial self-test or self-sample internet services.

### Next steps

A multifaceted stepped approach was developed during the workshop in how to proceed.

#### Development of a new roadmap

First of all, discussion should be promoted in guideline committees and in the professional community about the current scientific state of the art in benefits and harms of widespread testing of asymptomatic individuals. The viewpoint raised during this meeting suggests a paradigm shift and deviates from the ‘test and treat’ approach. International collaboration needs to be sought to reappraise the guidance on appropriate care and prevention in national and international guidelines. An update of the Cochrane review on screening for genital chlamydia^18^ as well as updated guidance on chlamydia control interventions from international organisations such as the European Centre for Disease Prevention and Control and WHO would be helpful.

#### Creation of an evidence-based narrative and description of knowledge gaps

Our viewpoint implies a different perception of a chlamydia infection, to be considered more as a common infection with uncommon severe complications than its portrayal as a silent killer of fertility. It raises the question if—and at what costs—every asymptomatic infection must be detected and must be treated. If chlamydia is considered as akin to *Mycoplasma genitalium*, the current approach to *M. genitalium*, that is—do not test unless symptomatic because widespread testing does more harm than good—needs to be critically reviewed also as a possible strategy for chlamydia. There are still critical knowledge gaps on the natural history of chlamydia infection. The consensus of the expert group was that the focus of research should be on determinants of complications rather than on determinants of infection. There is still a lack of controlled intervention studies that use clinical disease outcomes (in particular adverse fertility and pregnancy outcomes). Personalised and tailored clinical management and public health research can help to identify persons and populations who will benefit most without compromising the health of others.

#### Involve stakeholders and do not compromise sexual health

Care should be taken not to endanger the progress made in putting sexual health on the agenda, integrating prevention of STI, contraceptive services and positive attention for sexuality. Involvement of communities of youth, MSM and other stakeholders is therefore crucial in exploring future testing policies. Consideration should also be given to include diagnostic companies even though their market objectives are different. As a main reason for encounters with a sexual health professional for many individuals might be a request for a chlamydia or other STI tests, additional measures are warranted to prevent any negative impact on sexual healthcare seeking behaviour. Exploration of a bigger role for primary prevention is recommended.

#### Communicate and evaluate

Communicating wisely both in the professional field, as well as in the public domain, is imperative to facilitate shared decision taking and not to compromise faith in health authorities if different updated perspectives and guidelines are presented that deviate from earlier policies and practices. Given the areas of uncertainty surrounding a policy shift, monitoring and evaluation of any change of practices that take place will be essential. Surveillance systems should be in place to monitor any (adverse) consequences, including PID surveillance. A shift in chlamydia policy should not compromise testing of symptomatic persons, nor endanger early detection and treatment of other STIs like syphilis, gonorrhoea, HIV and hepatitis.

## Conclusion

The expert group’s consensus and interpretation of evidence was that chlamydia control is at crossroads. Opportunities for widespread testing are increasing, but the benefit–harm ratio of testing persons without symptoms is changing. Future strategies should shift their focus from *infection control*, promoting widespread testing, towards *disease management,* limiting and targeting testing, aiming at harm reduction.

**Key messages**

- To prevent late sequelae associated with chlamydia infection, widespread testing and treatment of asymptomatic persons to reduce the prevalence (‘test and treat paradigm’) have been implemented in routine practice in many high-income countries.
- Currently, there is no practice-based evidence that screening nor widespread chlamydia testing reduces the prevalence of chlamydia.
- The evidence that the ‘test and treat’ paradigm prevents chlamydia-associated health sequelae is absent for the prevention of tubal factor infertility and adverse pregnancy outcomes.
- Overdiagnosis and subsequent overtreatment contribute to the emergence of antimicrobial resistance in other pathogens and may harm the individual microbiome.
- The existing ‘test and treat’ paradigm to reduce chlamydia prevalence should be reconsidered.

10.1136/sextrans-2021-054992.supp3 Abstract translation This web only file has been produced by the BMJ Publishing Group from an electronic file supplied by the author(s) and has not been edited for content.

## References

[1] van Sighem A , Wit FWNM , Matser A . Monitoring report 2019. human immunodeficiency virus (HIV) infection in the Netherlands. Amsterdam: Stichting HIV monitoring, 2020. Available: www.hiv-monitoring.nl

[2] Staritsky, F van Aar LE , Visser M , et al . Sexually transmitted infections in the Netherlands 2019, Bilthoven, the Netherlands. RIVM 2020.

[3] David S , van Benthem B , Deug F , et al . Nationaal actio plan STI, HIV and sexual health 2017-2022. Bilthoven, the Netherlands. Available: https://rivm.openrepository.com/handle/10029/622149

[4] van Bergen J . Can we control Chlamydia? Keynote speech IUSTI-Europe, 2019. Available: https://pilv.lend.ee/iusti2019videogallery/#video25

[5] Public Health England . National Chlamydia screening programme external peer review: evidence pack. London: Public Health England, 2019.

[6] Van Der Pol B , Taylor SN , Mena L , et al . Evaluation of the performance of a point-of-care test for Chlamydia and gonorrhea. JAMA Netw Open 2020;3:e204819. 10.1001/jamanetworkopen.2020.4819 32407506 PMC7225902

[7] Wilson E , Leyrat C , Baraitser P , et al . Does internet-accessed STI (e-STI) testing increase testing uptake for Chlamydia and other STIs among a young population who have never tested? secondary analyses of data from a randomised controlled trial. Sex Transm Infect 2019;95:569–74. 10.1136/sextrans-2019-053992 31175210 PMC6902059

[8] Söderqvist J , Gullsby K , Stark L , et al . Internet-based self-sampling for Chlamydia trachomatis testing: a national evaluation in Sweden. Sex Transm Infect 2020;96:160–5. 10.1136/sextrans-2019-054256 31932359 PMC7231453

[9] van den Broek IVF , van Bergen JEAM , Brouwers EEHG , et al . Effectiveness of yearly, register based screening for Chlamydia in the Netherlands: controlled trial with randomised stepped wedge implementation. BMJ 2012;345:e4316. 10.1136/bmj.e4316 22767614 PMC3390168

[10] de Wit GA , Over EAB , Schmid BV , et al . Chlamydia screening is not cost-effective at low participation rates: evidence from a repeated register-based implementation study in the Netherlands. Sex Transm Infect 2015;91:423–9. 10.1136/sextrans-2014-051677 25759475

[11] Unemo M , Bradshaw CS , Hocking JS , et al . Sexually transmitted infections: challenges ahead. Lancet Infect Dis 2017;17:e235–79. 10.1016/S1473-3099(17)30310-9 28701272

[12] ECDC European Centre for Disease Prevention and Control . Chlamydia infection. in: ECDC. annual epidemiological report for 2018. Stockholm: ECDC, 2020. Available: https://www.ecdc.europa.eu/en/publications-data/chlamydia-infection-annual-epidemiological-report-2018

[13] Cassell JA , Low N . How can Chlamydia diagnoses increase when their complications are declining? Sex Transm Infect 2005;81:285–6. 10.1136/sti.2005.014902 16061531 PMC1745008

[14] Mitchell H , Allen H , Sonubi T . Mohammed H and contributors. sexually transmitted infections and screening for Chlamydia in England, 2019. London: Public Health England, 2020.

[15] Sonnenberg P , Clifton S , Beddows S , et al . Prevalence, risk factors, and uptake of interventions for sexually transmitted infections in Britain: findings from the National surveys of sexual attitudes and lifestyles (Natsal). Lancet 2013;382:1795–806. 10.1016/S0140-6736(13)61947-9 24286785 PMC3899025

[16] Hocking JS , Temple-Smith M , Guy R , et al . Population effectiveness of opportunistic Chlamydia testing in primary care in Australia: a cluster-randomised controlled trial. Lancet 2018;392:1413–22. 10.1016/S0140-6736(18)31816-6 30343857

[17] Price MJ , Ades AE , Soldan K , et al . The natural history of Chlamydia trachomatis infection in women: a multi-parameter evidence synthesis. Health Technol Assess 2016;20:1–250. 10.3310/hta20220 PMC4819202 27007215

[18] Low N , Redmond S , Uusküla A , et al . Screening for genital Chlamydia infection. Cochrane Database Syst Rev 2016;9:CD010866. 10.1002/14651858.CD010866.pub2 27623210 PMC6457643

[19] Davies B , Turner KME , Frølund M , et al . Risk of reproductive complications following Chlamydia testing: a population-based retrospective cohort study in Denmark. Lancet Infect Dis 2016;16:1057–64. 10.1016/S1473-3099(16)30092-5 27289389

[20] Schachter J , Chow JM . How can we improve outcomes of Chlamydia control programmes? Lancet Infect Dis 2016;16:989–90. 10.1016/S1473-3099(16)30131-1 27289390

[21] den Heijer CDJ , Hoebe CJPA , Driessen JHM , et al . Chlamydia trachomatis and the risk of pelvic inflammatory disease, ectopic pregnancy, and female infertility: a retrospective cohort study among primary care patients. Clin Infect Dis 2019;69:1517–25. 10.1093/cid/ciz429 31504315 PMC6792126

[22] Price MJ , Horner PJ , Ades AE . Risk of reproductive complications following Chlamydia testing. Lancet Infect Dis 2016;16:1223–4. 10.1016/S1473-3099(16)30379-6 27788979

[23] Hoenderboom BM , van Bergen JEAM , Dukers-Muijrers NHTM , et al . Pregnancies and time to pregnancy in women with and without a previous Chlamydia trachomatis infection. Sex Transm Dis 2020;47:739–47. 10.1097/OLQ.0000000000001247 32701764 PMC7553199

[24] Grant JS , Chico RM , Lee AC , et al . Sexually transmitted infections in pregnancy: a narrative review of the global research gaps, challenges, and opportunities. Sex Transm Dis 2020;47:779–89. 10.1097/OLQ.0000000000001258 32773611 PMC7668326

[25] Tang W , Mao J , Li KT , et al . Pregnancy and fertility-related adverse outcomes associated with Chlamydia trachomatis infection: a global systematic review and meta-analysis. Sex Transm Infect 2020;96:322–9. 10.1136/sextrans-2019-053999 31836678 PMC7292777

[26] US Preventive Services Task Force, Owens DK , Davidson KW , et al . Screening for bacterial vaginosis in pregnant persons to prevent preterm delivery: US preventive services Task force recommendation statement. JAMA 2020;323:1286–92. 10.1001/jama.2020.2684 32259236

[27] van Wees DA , Drissen MMCM , den Daas C , et al . The impact of STI test results and face-to-face consultations on subsequent behavior and psychological characteristics. Prev Med 2020;139:106200. 10.1016/j.ypmed.2020.106200 32659244

[28] Soetens LC , van Benthem BHB , Op de Coul ELM . Chlamydia test results were associated with sexual risk behavior change among participants of the Chlamydia screening implementation in the Netherlands. Sex Transm Dis 2015;42:109–14. 10.1097/OLQ.0000000000000234 25668640

[29] Batteiger BE , Xu F , Johnson RE , et al . Protective immunity to Chlamydia trachomatis genital infection: evidence from human studies. J Infect Dis 2010;201 Suppl 2:178–89. 10.1086/652400 20524235 PMC2990949

[30] Omori R , Chemaitelly H , Althaus CL , et al . Does infection with Chlamydia trachomatis induce long-lasting partial immunity? Insights from mathematical modelling. Sex Transm Infect 2019;95:115–21. 10.1136/sextrans-2018-053543 30181327 PMC6580764

[31] Brodersen J , Schwartz LM , Heneghan C , et al . Overdiagnosis: what it is and what it isn't. BMJ Evid Based Med 2018;23:1–3. 10.1136/ebmed-2017-110886 29367314

[32] Welch HG , LM lmscharz , Woloshin S . Overdiagnosed: making people sick in the pursuit of health. Kindler 1th edition isbn-10 0807022004.

[33] Molano M , Meijer CJLM , Weiderpass E , et al . The natural course of Chlamydia trachomatis infection in asymptomatic Colombian women: a 5-year follow-up study. J Infect Dis 2005;191:907–16. 10.1086/428287 15717266

[34] Dukers-Muijrers NHTM , Wolffs P , Lucchesi M , et al . Oropharyngeal Chlamydia trachomatis in women; spontaneous clearance and cure after treatment (FemCure). Sex Transm Infect 2021;97:147–51. 10.1136/sextrans-2020-054558 32737209

[35] Rank RG , Yeruva L . An alternative scenario to explain rectal positivity in Chlamydia-infected individuals. Clin Infect Dis 2015;60:1585–6. 10.1093/cid/civ079 25648236

[36] Dukers-Muijrers NHTM , Janssen KJH , Hoebe CJPA , et al . Spontaneous clearance of Chlamydia trachomatis accounting for bacterial viability in vaginally or rectally infected women (FemCure). Sex Transm Infect 2020;96:541–8. 10.1136/sextrans-2019-054267 32066588

[37] Janssen KJH , Wolffs P , Lucchesi M , et al . Assessment of rectal Chlamydia trachomatis viable load in women by viability-PCR. Sex Transm Infect 2020;96:85–8. 10.1136/sextrans-2019-054002 31383780

[38] Kenyon CR , Schwartz IS . Effects of sexual network connectivity and antimicrobial drug use on antimicrobial resistance in Neisseria gonorrhoeae. Emerg Infect Dis 2018;24:1195–203. 10.3201/eid2407.172104 29912682 PMC6038757

[39] van de Wijgert JHHM . The vaginal microbiome and sexually transmitted infections are interlinked: consequences for treatment and prevention. PLoS Med 2017;14:e1002478. 10.1371/journal.pmed.1002478 29281632 PMC5744905

