# Supplementary material for: Where to go to in chlamydia control? From infection control towards infectious disease control
Source: Sex Transm Infect. 2021 May 27;97(7):501–6. doi: 10.1136/sextrans-2021-054992 (PMC8543211; doi:10.1136/sextrans-2021-054992)
Supplement: Supplementary data [file sextrans-2021-054992supp001.pdf]

Rijksinstituut voor Volksgezondheid en Milieu
Ministerie van Volksgezondheid,

Invitational workshop Chlamydia (s)Top 8 November 2019 Utrecht, The Netherlands

PROJECT TITLE: The future of Chlamydia control in the Netherlands

PROJECT SUBTITLE: Time to change paradigms?

PROJECT SUBJECT: This 1-day invitational workshop will bring together a range of national and international experts to discuss the state-of-art in Chlamydia control and to explore recommendations for future avenues in the Netherlands.

PROJECT JUSTIFICATION: The overall purpose of this workshop is to redefine the benefits and harms of current test-and-treat strategies in Chlamydia control. The state of science and practice suggests that we are at cross-roads between more and more testing on one hand- including screening and upscaling extragenital testing-, and downscaling/ de-implementing / de-investing our test-activities on the other hand.

PROJECT BACKGROUND: New developments in POC tests and, mobile health offer accelerated opportunities to detect and treat infections. But the evidence that we are doing more good than harm with accelerated testing is under debate. The low complication rate of long term sequelae contribute to a high Number Unnecessary Treated (NUT) and the limited attributable fraction of CT infections in PID and Tubal Factor Infertility (TFI) challenge cost-effectivity of screening. Moreover there is no practice based evidence that a sustained uptake of screening is feasible, and that sizeable reductions in chlamydia prevalence can be realized by screening.
Accelerated testing also introduces harms, including overdiagnosis, overtreatment, antimicrobial resistance issues for pathogens other than Chlamydia, and changes in the microbioma.
Balancing benefit and harms is very timely .

PROJECT AIMS:

1. To discuss the current equipoise on Chlamydia screening and reflect on the evolving land-scape of Chlamydia control interventions, including accelerated case-finding and extragenital testing.
2. To (re-)appraise the benefits and harms of accelerated case-finding of asymptomatic *Chla-mydia trachomatis* (CT) infections
3. To discuss the way ahead: up-scaling or downscaling detection of asymptomatic CT infec-tions (Strengths, Weaknesses, Opportunities, Threats-SWOT)

Chlamydia control workshop 08/11/2019

4. To explore new paradigms for CT *infectious disease* control considering latest scientific developments
5. To formulate recommendations for future policies and practices, including research and monitoring

WORKSHOP OUTCOME:
- Report of the meeting/paper
- Updated viewpoint
- Recommendations to develop future strategies for CT control in the Netherlands as indicated in the National Action Plan for sexual Health 2017-2022

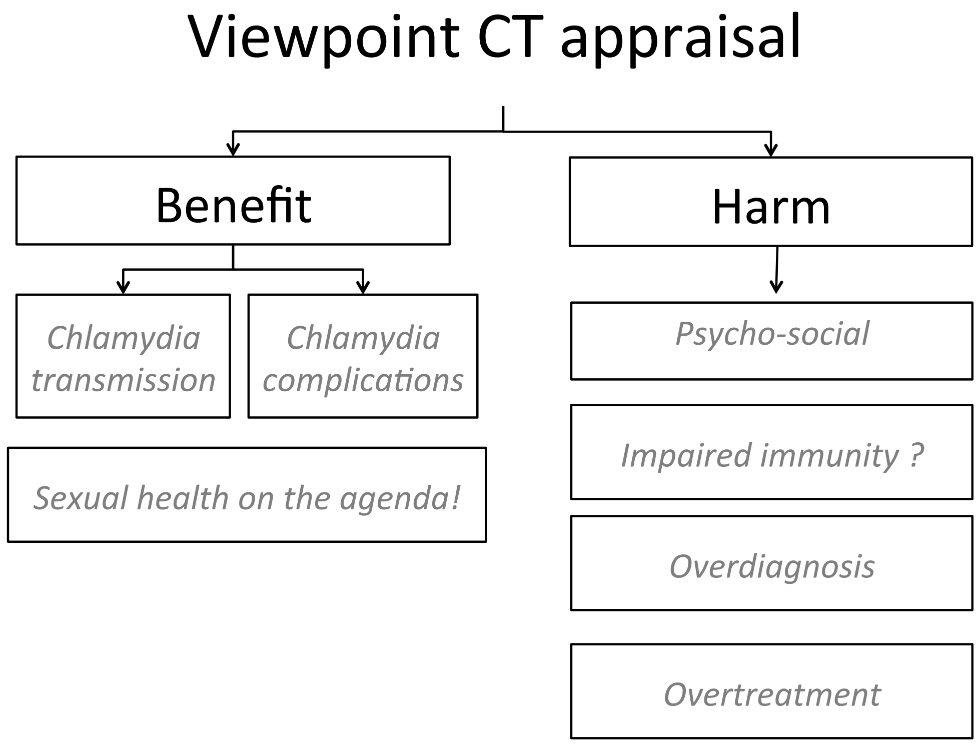

Chlamydia control workshop 08/11/2019

WORKSHOP STRUCTURE:

09.00-09.30: Coffee- tea
09.30-09.50: welcome and introduction.
Febe Deug (chair-person) , Silke David, Mark Vermeulen,

Setting the stage
09.50 - 10.10: Changing paradigms: from infection control to *infectious disease* control. Jan van Bergen
10.10 - 10.30: Chlamydia control policies and monitoring in the Netherlands. Birgit van Benthem
10.30 - 11.00: Personal communications
a. UK NCSP and the way forward - Helen Ward
b. Cochrane revisited (incl. ACCEPT trial Australia) and the Swiss way - Nicola Low
c. Changing perspectives in Sweden - Bjorn Herrmann
11.00 – 11.15 Coffee – tea break

Finding the balance
Quote: *"Should I treat chlamydia as a major pathogen amidst a range of STIs, causing reproductive health events and reduced sexual health or is the bacterium mostly commensal, part of the microbiome with only occasional adverse outcomes?"*

11.15 – 12.45: Discussion: harms and benefits of accelerated testing asymptomatics in relation to 3 specific groups:
- heterosexual at risk/young people (incl. extragenital)
- MSM
- Pregnant women
: Method: Put your weight in the scale (line-up/add and remove harms/benefits)
: Outcome: (extended) viewpoint and preliminary conclusions
12.45 – 14.00: Lunch

Where to go?
Quote: "*Should we manage CT control like we do now with Mycoplasma genitalium where we only test symptomatics?"*

14.00 – 15.30: Discussion: Scale up or scale down?
: Method: SWOT in relation to the 3 specific groups,.
: Outcome: position statement including risk-assesment
15.30-15.45: tea-break

15.45 – 17.15: Discussion: future options and recommendations
:Method: discussion
:Outcome: roadmap
17.15 – 17. 30: Closure

18.00: Dinner

Chlamydia control workshop 08/11/2019

LIST OF PROJECT PARTICIPANTS

1. Dr. Fleur van Aar (RIVM, the Netherlands)
2. Dr. Birgit van Benthem (RIVM, the Netherlands)
3. Prof. Jan van Bergen (University of Amsterdam, the Netherlands)
4. Drs. Hanna Bos (Soa Aids Nederland)
5. Dr. Silke David(RIVM, the Netherlands
6. Drs. Febe Deug (Soa Aids Nederland)
7. Dr. Nicole Dukers-Muijrers (GGD Zuid-Limburg, the Netherlands)
8. Prof. Christian Hoebe (GGD Zuid-Limburg, the Netherlands)
9. Dr. Hannelore Götz (GGD Rotterdam-Rijnmond, the Netherlands)
10. Prof. Nicola Low (University of Berne, Switzerland)
11. Prof. Servaas Morré (VUmc, Amsterdam, the Netherlands)
12. Prof Bjorn Herrmann (Uppsala University, Sweden)
13. Dr. Janneke Heijne (RIVM, the Netherlands)
14. Drs. Bernice Hoenderboom (RIVM, the Netherlands)
15. Prof. Marianne van der Sande (Instituut of Tropical Medicine, Antwerp, Belgium)
16. Dr . Gianfranco Spiteri (ECDC Sweden)
17. Prof. Henry de Vries (GGD Amsterdam, the Netherlands)
18. Prof.Helen Ward (Imperial College London, UK)

Chlamydia control workshop 08/11/2019
