# Supplementary material for: Where to go to in chlamydia control? From infection control towards infectious disease control
Source: Sex Transm Infect. 2021 May 27;97(7):501–6. doi: 10.1136/sextrans-2021-054992 (PMC8543211; doi:10.1136/sextrans-2021-054992)
Supplement: Supplementary data [file sextrans-2021-054992supp002.pdf]

Declaration

I, the Submitting Author has the right to grant and does grant on behalf of all authors of the Work (as defined in the below author licence), an exclusive licence and/or a non-exclusive licence for contributions from authors who are: i) UK Crown employees; ii) where BMJ has agreed a CC-BY licence shall apply, and/or iii) in accordance with the terms applicable for US Federal Government officers or employees acting as part of their official duties; on a worldwide, perpetual, irrevocable, royalty-free basis to BMJ Publishing Group Ltd ("BMJ") its licensees and where the relevant Journal is co-owned by BMJ to the co-owners of the Journal, to publish the Work in Sexually Transmitted Infections and any other BMJ products and to exploit all rights, as set out in our licence.

Jan van Bergen, Amsterdam 21-04-2021

van Bergen JEAM, *et al. Sex Transm Infect* 2021; 97:501–506. doi: 10.1136/sextrans-2021-054992
