# Supplementary material for: Where to go to in chlamydia control? From infection control towards infectious disease control
Source: Sex Transm Infect. 2021 May 27;97(7):501–6. doi: 10.1136/sextrans-2021-054992 (PMC8543211; doi:10.1136/sextrans-2021-054992)
Supplement: Abstract translation [file sextrans-2021-054992supp003.pdf]

Korte Nederlandse samenvatting

Doel: Inzicht genereren in de richting van de toekomstige chlamydia bestrijding in Nederland en met name in het al dan niet nastreven van actieve opsporing van asymptomatische genitale en extra-genitale chlamydia infecties.

Methode: literatuuronderzoek en besloten workshop met nationale en internationale chlamydia experts.

Resultaten: proactief opsporen van asymptomatische chlamydia infecties beoogt verdere verspreiding tegen te gaan en complicaties te voorkomen. Onderzoeken laten echter geen bewijs zien dat de populatie-prevalentie wordt teruggebracht door screening en/of door routinematig testen op asymptomatische infecties. Er is beperkt bewijs van matige kwaliteit dat het risico op PID door screening kan worden verminderd. De kans op tubaire infertiliteit is laag en er ontbreekt wetenschappelijk bewijs of en in welke mate tubaire infertiliteit kan worden verhinderd of verminderd met wijdverspreid testen. Over-diagnostiek van infecties die merendeels vanzelf overgaan, en waarvan een deel '*non-viable*' blijkt te zijn, leidt tot overbehandeling met antibiotica. Overbehandeling heeft als gevolg antibiotica-resistentie en aantasting van het oraal, anaal en genitaal microbioom. Veranderende inzichten zullen uitkomsten van eerdere kost-effectiviteit studies in een ander daglicht plaatsen.

Conclusie: de balans tussen voor- en nadelen van wijdverbreid testen op asymptomatische chlamydia infecties is aan het schuiven. De expert-groep stelt het huidige wijdverspreid "test-en-behandel" paradigma ter discussie en beveelt eerder het verminderen dan het uitbreiden van testen op asymptomatische chlamydia infecties aan.
